# Influence of ensitrelvir or nirmatrelvir/ritonavir on tacrolimus clearance in kidney transplant recipients: a single-center case series

**DOI:** 10.1186/s40780-024-00361-x

**Published:** 2024-07-10

**Authors:** Hanako Naganawa, Yoshiki Katada, Shunsaku Nakagawa, Keisuke Umemura, Hiroki Ishimura, Moto Kajiwara, Hiroki Endo, Mitsuhiro Sugimoto, Yurie Katsube, Kinuka Kotani, Saki Ohta, Daiki Hira, Masahiro Tsuda, Yuki Kita, Takashi Kobayashi, Tomohiro Terada

**Affiliations:** 1 Department of Clinical Pharmacology and Therapeutics, Kyoto University Hospital, 54 Shogoin- Kawahara-cho, Sakyo-ku, Kyoto, 606-8507 Japan; 2 Graduate School of Pharmaceutical Sciences, Kyoto University, 46-29 Yoshida-Shimo-Adachi-cho, Sakyo-ku, Kyoto, 606-8501 Japan; 3 Department of Urology, Graduate School of Medicine, Kyoto University, 54 Shogoin- Kawahara-cho, Sakyo-ku, Kyoto, 606-8507 Japan

**Keywords:** Drug–drug interaction, Tacrolimus, Ensitrelvir, Nirmatrelvir/ritonavir, COVID-19, Kidney transplantation

## Abstract

**Background:**

Among the oral antivirals used for treating patients with mild-to-moderate novel coronavirus disease 2019 (COVID-19), nirmatrelvir/ritonavir (NMV/RTV) and ensitrelvir (ESV) are inhibitors of cytochrome P450 (CYP) 3A, and therefore, can cause drug–drug interactions with concomitant medications. Tacrolimus (TAC), a substrate of CYP3A4/5, is administered for a long period to prevent rejection after kidney transplantation. TAC should be discontinued while using NMV/RTV because blood TAC levels significantly increase when these drugs are concomitantly administered. However, the influence of ESV on blood TAC levels has not yet been reported, and the management of TAC doses during the use of ESV remains unclear.

**Case presentation:**

We experienced three kidney transplant recipients with COVID-19, whose blood trough levels of TAC increased by the concomitant use of NMV/RTV or ESV. In two patients administering NMV/RTV, blood trough levels of TAC increased more than tenfold after combination therapy, whereas in one patient administering ESV, TAC level increased approximately threefold.

**Conclusions:**

These cases suggest that TAC administration should be discontinued during NMV/RTV treatment to maintain blood TAC levels within the therapeutic range, and a reduced TAC dose is sufficient during ESV treatment.

## Background

Solid-organ transplant recipients are at high risk of severe coronavirus disease 2019 (COVID-19) as they undergo long-term immunosuppressive therapy [1]. Nirmatrelvir/ritonavir (NMV/RTV) is the first oral drug for COVID-19 approved in Japan, and RTV is a potent inhibitor of cytochrome P450 (CYP) 3A [2]. Therefore, scrutinizing drug–drug interactions during administration of concomitant medications is necessary. Ensitrelvir (ESV) has been approved for treating patients with mild-to-moderate symptoms of COVID-19 [3–5]. This drug has been available in Japan and some other countries as of March 2024. As ESV is also a potent inhibitor of CYP3A [6], drug–drug interactions with the substrates for CYP3A should be monitored when administering ESV. In particular, solid organ transplant patients commonly take tacrolimus (TAC) that is a substrate for CYP3A4/5; therefore, predicting the alterations in clearance of TAC and managing its doses are necessary during concomitant administration of CYP3A inhibitors [7].

Regarding drug–drug interactions between NMV/RTV and TAC, studies have reported 5–15-fold or higher increases in blood trough levels of TAC [8, 9] and related serious adverse reactions [8–11]. Therefore, some reports recommend that TAC should be discontinued while administering NMV/RTV [10–12]. However, as for ESV, clinical recommendations for managing drug–drug interactions of TAC are not available, and how to adjust TAC dosage during concomitant use of ESV and TAC remains unclear.

Here we present three cases of COVID-19 after kidney transplantation. One patient received ESV, while the others were treated with NMV/RTV. This report describes the fluctuations in blood TAC levels during treatment, and discusses their management strategies.

## Case presentation

### Case 1

**Fig. 1:**
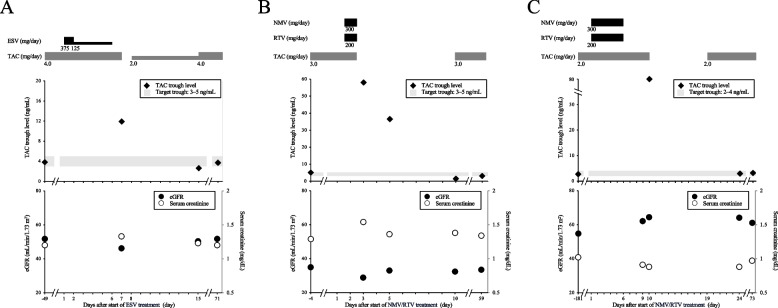
Clinical course of cases 1, 2, and 3. Dosages of ESV, NMV/RTV, and TAC, TAC trough levels, and renal function are shown. **A** Case 1, **B** Case 2, and **C** Case 3. ESV, ensitrelvir; NMV/RTV, nirmatrelvir/ritonavir; TAC, tacrolimus; eGFR, estimated glomerular filtration rate

A Japanese male (height, 162.0 cm; weight 58.0 kg) in his 50 s underwent living-donor kidney transplantation for focal glomerulosclerosis and was followed-up at our hospital for 25 years. The patient had a history of hypercholesterolemia. Table 1 lists the baseline characteristics and medications administered to him. The daily dose of TAC was 4.0 mg, and blood trough level was stable at 3–5 ng/mL. Genetic polymorphism of *CYP3A5* in this patient was *1/*3. He developed fever, and his primary care physician diagnosed him with COVID-19 (day 1). The physician prescribed ESV (375 mg on day 1 and 125 mg once daily from days 2 to 5). TAC administration was continued throughout the period of ESV treatment. On day 7, the patient visited our hospital for regular follow-up, and blood trough level of TAC was found to be elevated to 11.9 ng/mL. No adverse effects of TAC, such as headache, elevated blood pressure, or renal dysfunction, were observed. Renal dysfunction was defined as an increase in serum creatinine of > 0.3 mg/L or 50% from baseline. On day 7, TAC administration was stopped for 1 day and resumed at 2.0 mg/day from day 8. On day 15, blood trough level of TAC decreased to 2.6 ng/mL, and TAC dose was reinstated to 4.0 mg/day. No rejection was observed during this period, and his COVID-19 infection was cured. The clinical course of ESV and TAC doses, blood trough levels of TAC, and renal function are shown in Fig. 1A. Since renal dysfunction is a common side effect of TAC, trends in renal function were monitored throughout the study.

**Table 1:** Baseline characteristics, medications, and daily doses in case 1

Variable	Patient 1
**Characteristics**	
Age (years)	50s
Gender	Male
Height (cm)	162.0
Weight (kg)	58.0
* CYP3A5* genotype	*1/*3
**Medication**	**Daily dosage**
Tacrolimus	4 mg/day
Prednisolone	5 mg/day
Azathioprine	50 mg/day
Benzbromarone	12.5 mg/day
Atorvastatin	5 mg/day
Ambroxol	45 mg/day
Lactomin	2 g/day

### Case 2

A Japanese female (height, 162.7 cm; weight 54.6 kg) underwent living-donor kidney transplantation for immunoglobulin A in her 50 s. After 9 years, the patient was diagnosed with COVID-19. She had a history of breast cancer, adenomyosis, and dysmenorrhea. Her baseline characteristics and medications are listed in Table 2. The patient was administering 3.0 mg/day TAC as an immunosuppressant, and its blood trough level was stable at 3–5 ng/mL. Genetic polymorphism of *CYP3A5* in this patient was *3/*3. Her primary care physician diagnosed her with COVID-19 (day 1) and prescribed NMV/RTV. The patient started administreing NMV/RTV and consulted her transplant coordinator at our institution on day 2 to determine whether NMV/RTV and TAC could be administered in combination. On day 2, her transplant physician at our hospital instructed her to discontinue both TAC and NMV/RTV because of concerns regarding drug–drug interaction. On day 3, the patient experienced diarrhea, vomiting, headache, and pharyngeal pain, and visited our hospital. Blood trough level of TAC was 58 ng/mL on day 3. From days − 4 to 3, serum creatinine level increased from 1.29 to 1.54 mg/dL, and the estimated glomerular filtration rate decreased from 34.9 to 28.8 mL/min/1.73 m^2^. From day 3, molnupiravir, which does not inhibit CYP3A, was administered instead of NMV/RTV for treating COVID-19. On day 5, blood trough level of TAC was 36.5 ng/mL, which exceeded the upper limit of the target range although TAC had been discontinued. On day 10, blood trough level of TAC decreased to 1.5 ng/mL, and TAC administration was resumed at 3.0 mg/day. No rejection was observed during this period, and her COVID-19 infection was cured. The clinical course of NMV/RTV and TAC doses, blood trough levels of TAC, and renal function are shown in Fig. 1B.

**Table 2:** Baseline characteristics, medications, and daily doses in case 2

Variable	Patient 2
**Characteristics**	
Age (years)	50s
Gender	Female
Height (cm)	162.7
Weight (kg)	54.6
* CYP3A5* genotype	*3/*3
**Medication**	**Daily dosage**
Tacrolimus	3 mg/day
Mycophenolate mofetil	1000 mg/day
Prednisolone	5 mg/day
Rabeprazole	10 mg/day
Atorvastatin	5 mg/day
Alfacalcidol	0.5 µg/day
Telmisartan	40 mg/day
Amlodipine	5 mg/day
Dried ferrous sulfate	105 mg/day
Estradiol	1 mg/day
Progesterone	100 mg/day

### Case 3

A Japanese male (height, 164.5 cm; weight 59.2 kg) in his 70 s underwent deceased-donor kidney transplantation 20 years ago. The patient had a history of hypertension, and the baseline characteristics and medications are listed in Table 3. He was administering 2.0 mg/day TAC as an immunosuppressant, and blood trough level of TAC was stable at 2–4 ng/mL. Genetic polymorphism of *CYP3A5* in this patient was *3/*3. He visited his primary care physician because of fever and was diagnosed with COVID-19 (day 1). The physician prescribed NMV/RTV. TAC was continued during NMV/RTV treatment. The patient visited our hospital for a scheduled follow-up on day 10, and blood trough level of TAC was abnormally high (> 80 ng/mL), which was higher than the upper limit of quantification. No adverse effects of TAC, such as headache, elevated blood pressure, or renal dysfunction, were observed. TAC was discontinued from days 10 to 18, and resumed at 2.0 mg/day from day 19. On day 24, blood trough level of TAC was 2.9 ng/mL, which was within the target range. Therefore, TAC was continued at 2.0 mg/day. No rejection was observed during this period, and his COVID-19 infection was cured. The clinical course of NMV/RTV, TAC dosage, blood trough levels of TAC, and renal function are shown in Fig. 1C.

**Table 3:** Baseline characteristics, medications, and daily doses in case 3

Variable	Patient 3
**Characteristics**	
Age (years)	70s
Gender	Male
Height (cm)	164.5
Weight (kg)	59.2
* CYP3A5* genotype	*3/*3
**Medication**	**Daily dosage**
Tacrolimus	2 mg/day
Mycophenolate mofetil	500 mg/day
Prednisolone	2.5 mg/day
Amlodipine	2.5 mg/day

In all three cases, no adverse events were observed, including renal dysfunction, a typical side effect of TAC, or other adverse events, such as central nervous system disorders, cerebrovascular disorders, thrombotic microangiopathy, or infections. In addition, laboratory parameters other than renal function associated with adverse events of TAC and interactions were within normal limits in these cases (Table 4).

**Table 4:** Changes in laboratory parameters before and after increases in blood trough levels of TAC in cases 1–3

Parameters	Normal range	Case 1			Case 2			Case 3		
		Before	Onset	After	Before	Onset	After	Before	Onset	After
		Day-48	Day7	Day15	Day-6	Day3	Day8	Day-53	Day10	Day24
Hemoglobin (g/dL)	11.6–14.8	15.1	14.4	13.9	11.4	14	12.8	13.4	13.8	12.8
Hematocrit (%)	35.1–44.4	45.4	45.4	42.7	37.4	42.2	38.6	42.6	41.3	39.1
Aspartate aminotransferase (U/L)	13–40	21	24	18	14	22	13	21	27	21
Alanine aminotransferase (U/L)	10–42	14	20	15	14	27	14	13	42	20
Total bilirubin (mg/dL)	0.4–1.5	0.8	0.7	0.7	0.8	0.6	0.7	1.0	0.9	0.5
Urea nitrogen (mg/dL)	8–20	23	29	29	21	19	25	18	21	15
Sodium (mmol/L)	138–145	141	142	141	143	137	142	139	134	140
Potassium (mmol/L)	3.6–4.8	3.9	4.3	3.9	4.0	3.7	4.1	4.0	4.4	4.1
Chloride (mmol/L)	101–108	107	107	107	106	102	106	103	99	104
Magnesium (mg/dL)	1.8–2.4	-	-	-	1.9	1.6	1.8	-	-	-

## Discussion and conclusions

Kidney-transplant recipients require TAC throughout their lives, and unstable blood trough levels of TAC increase the risk of graft rejection and infection [13, 14]. Therefore, managing drug–drug interactions between TAC and concomitant medications is crucial. We encountered one case, in which ESV treatment increased blood trough levels of TAC by approximately threefold; in two other cases, NMV/RTV treatment increased blood trough levels of TAC by more than tenfold. Although both ESV and NMV/RTV are potent inhibitors of CYP3A, the influence of ESV on blood TAC levels was lower than that of NMV/RTV. These results suggest that different approaches are necessary for maintaining blood TAC levels when ESV and NMV/RTV are concomitantly administered.

ESV and NMV/RTV inhibit CYP3A [2, 6]. The inhibition ratio of CYP3A by ESV is approximately 0.93 [6, 15, 16], which is a strong CYP3A-inhibitory effect similar to that of itraconazole [16]. The concentration/dose ratio of TAC increases 2.7-fold when co-administered with itraconazole [17]. In our case 1, TAC levels increased approximately threefold, suggesting that the degree of interaction between ESV and TAC was comparable to that between itraconazole and TAC. As ESV has a long half-life of 51.4 h [18], its CYP3A-inhibitory effect is expected to continue for approximately 1–2 weeks after discontinuation of ESV administration. This assumption is roughly consistent with the clinical course in case 1. In the three cases of the present study, the trend of changes in TAC concentrations differed between ESV and NMV/RTV treatments, indicating the influence of CYP3A as well as P-glycoprotein. TAC has poor intestinal bioavailability and a high potential for strong interactions with P-glycoprotein inhibitors. The mechanism of interaction between NMV and TAC involves inhibition of CYP3A and P-glycoprotein in the small intestine, as well as systemic hepatic metabolism by CYP3A [19]. Both RTV and ESV inhibit P-glycoprotein, but the degree of inhibition may vary. Despite the limitations of a single case of ESV treatment in the present report, we suggest the following strategies: 1) The TAC dose could be reduced to at least 33% at the start of ESV administration; 2) consider measuring blood TAC levels to determine whether the interaction persists for at least a week after discontinuation of ESV administration; and 3) TAC doses should be adjusted accordingly. Due to the single case and limited types of the *CYP3A5* genetic polymorphisms, further studies are required to determine the extent of the drug interaction between TAC and ESV.

Among the clinically used drugs, RTV is the most potent inhibitor of CYP3A. Therefore, RTV has been applied as a booster for drugs with low oral bioavailability owing to CYP3A-mediated metabolism [20]. Prikis et al. have reported the case of a kidney transplant patient with COVID-19, in which the blood trough level of TAC was maintained at 4–6 ng/mL; however, it exceeded 30 ng/mL after 2 days of NMV/RTV administration. Serum creatinine level increased from 1.42 to 1.79 mg/dL [9]. It was also reported in a case with liver transplantation whose blood trough levels of TAC increased from 3.6–6.0 ng/mL to > 60 ng/mL after 4 days of NMV/RTV administration [11]. According to previous reports [8–12], TAC should be discontinued during NMV/RTV administration because maintaining blood TAC levels within the therapeutic range is difficult during these concomitant treatments.

In the present study, increases in blood trough levels of TAC after concomitant NMV/RTV administration were slightly higher with 10–20-fold or more increases than that in previous reports with 5–15-fold or more increases [8, 9, 11]. TAC is metabolized by CYP3A4 and CYP3A5 [21]. In kidney transplant patients, genetic polymorphisms of *CYP3A5* affect blood trough levels of TAC, whereas genetic polymorphisms of *CYP3A4* have no effect on blood trough levels of TAC [22]. Although genetic polymorphism of *CYP3A5* has not been tested in previous case reports [8, 9, 11], the present cases 2 and 3 had the genotype CYP3A5 *3/*3, indicating the loss of function of CYP3A5. Chandel et al. have reported that dose-adjusted trough concentrations of TAC increased in all *CYP3A5* genotypes with concurrent use of ketoconazole, a strong inhibitor of CYP3A4 and CYP3A5, and the magnitude of the increase from baseline in CYP3A5*3/*3 patients was higher than that in CYP3A5*1 carriers [23]. It has also reported that the combination of TAC with itraconazole in CYP3A5*3/*3 patients lead to a higher risk of high TAC exposure [24]. Because RTV potently inhibits CYP3A4 and CYP3A5, the intensity of the interaction may be greater when NMV/RTV is co-administered in patients with CYP3A5*3/*3. Conversely, Tomida et al. have reported the intensity of interaction with the concomitant use of NMV/RTV might be stronger in *CYP3A5* *1 carriers, who would have relatively lower bioavailability and high clearance of TAC [19]. Although TAC concentrations increased in the present cases, the effect of CYP3A5 genetic polymorphisms on the drug interaction between TAC and RTV has not been reported; further studies are required. In contrast, the genetic polymorphisms of *CYP3A5* in the case with ESV (case 1) were *1/*3. Therefore, if the genetic polymorphisms of *CYP3A5* is *1/*1 or *3/*3, the degree of interaction may differ from that observed in this case. Future pharmacokinetic analyses taking into account *CYP3A5* genetic polymorphisms are necessary for validating dosage adjustment strategies when NMV/RTV is administered in combination with TAC.

Considering these cases, failure to adjust TAC (through drug withdrawal or dose reduction) while administering ESV or NMV/RTV is a serious issue that warrant attention. Given that primary care physicians and community pharmacists frequently deal with COVID-19 in clinics, it is essential for them to be aware of these drug–drug interactions. Therefore, disseminating and implementing guidelines on drug interactions in the treatment of COVID-19 is necessary. Although the package inserts for NMV/RTV have been revised in response to previous reports, there is a need for further public awareness. Efforts should be made to ensure that this information is effectively communicated to primary care physicians, community pharmacists, and pharmaceutical companies.

Kidney transplant recipients are at high risk of severe COVID-19 infection because they undergo long-term immunosuppressive therapy. Both ESV and NMV/RTV, which are oral medications of COVID-19, are CYP3A inhibitors, and their interactions with TAC should be monitored. When ESV is administered, the TAC blood trough concentration can be controlled by reducing the TAC dose. In contrast, discontinuation of TAC is recommended when initiating NMV/RTV treatment. This is the first clinical report on the interaction between TAC and ESV. The concentration trends and clinical course of TAC combined with ESV are different from those with NMV/RTV.

## Data Availability

Data used in this case report will not be shared owing to the risk of identifying the individuals.

## Abbreviations

COVID-19: Coronavirus disease 2019
CYP: Cytochrome P450
ESV: Ensitrelvir
NMV/RTV: Nirmatrelvir/ritonavir
TAC: Tacrolimus
